# Optical Imaging of Neuronal Activity and Visualization of Fine Neural Structures in Non-Desheathed Nervous Systems

**DOI:** 10.1371/journal.pone.0103459

**Published:** 2014-07-25

**Authors:** Christopher John Goldsmith, Carola Städele, Wolfgang Stein

**Affiliations:** 1 School of Biological Sciences, Illinois State University, Normal, Illinois, United States of America; 2 Institute of Neurobiology, Ulm University, Ulm, Germany; Claremont Colleges, United States of America

## Abstract

Locating circuit neurons and recording from them with single-cell resolution is a prerequisite for studying neural circuits. Determining neuron location can be challenging even in small nervous systems because neurons are densely packed, found in different layers, and are often covered by ganglion and nerve sheaths that impede access for recording electrodes and neuronal markers. We revisited the voltage-sensitive dye RH795 for its ability to stain and record neurons through the ganglion sheath. Bath-application of RH795 stained neuronal membranes in cricket, earthworm and crab ganglia *without* removing the ganglion sheath, revealing neuron cell body locations in different ganglion layers. Using the pyloric and gastric mill central pattern generating neurons in the stomatogastric ganglion (STG) of the crab, *Cancer borealis*, we found that RH795 permeated the ganglion without major residue in the sheath and brightly stained somatic, axonal and dendritic membranes. Visibility improved significantly in comparison to unstained ganglia, allowing the identification of somata location and number of most STG neurons. RH795 also stained axons and varicosities in non-desheathed nerves, and it revealed the location of sensory cell bodies in peripheral nerves. Importantly, the spike activity of the sensory neuron AGR, which influences the STG motor patterns, remained unaffected by RH795, while desheathing caused significant changes in AGR activity. With respect to recording neural activity, RH795 allowed us to optically record membrane potential changes of sub-sheath neuronal membranes without impairing sensory activity. The signal-to-noise ratio was comparable with that previously observed in desheathed preparations and sufficiently high to identify neurons in single-sweep recordings and synaptic events after spike-triggered averaging. In conclusion, RH795 enabled staining and optical recording of neurons through the ganglion sheath and is therefore both a good anatomical marker for living neural tissue and a promising tool for studying neural activity of an entire network with single-cell resolution.

## Introduction

An important prerequisite for studying the properties and connectivity of neural circuits is to locate the same neuron or classes of neurons in each preparation. However, even in nervous systems with a small number of neurons, determining neuron location can be challenging because neurons are densely packed and found in different layers, and the nervous system is often covered by a protective sheath. Another challenge in identifying circuits is to simultaneously record the activities of the circuit neurons. Besides field potential recordings and the use of multi-electrode arrays, optical imaging with either expressed or bath-applied calcium- or voltage-indicators is often the preferred method (for reviews see [1]–[3]). Yet, in systems where expression is not possible the success of the dye application is impeded by the presence of a ganglion sheath or connective tissue that prevents dye permeability [4]. Consequently, tissue slices are taken, or the ganglion sheath and connective tissue are removed by microsurgery or enzymatic treatment before dye application, procedures both difficult and potentially harmful to the neurons [5]–[7]. In the thoracic ganglia of the phasmid *Extatosoma tiaratum*, for example, surgically removing the sheath results in a loss of hydrostatic pressure inside the ganglion and a bulging out of the neurons [8]. Even when desheathing is possible, it is time-intensive, requires specific protocols (enzymatic approach) and/or many months of training in microsurgery [6], [9]–[11]. There is always the potential of damaging neurons, leading to a rather significant failure rate of the desheathing process in some systems. Even when the procedure is successful, the influence of removing the sheath on the neural activity is often unclear because a direct comparison of neural activity before and after desheathing is missing. This is despite the fact that there is evidence that the ganglion sheath can affect interstitial voltage and ion concentrations [12].

Here, we are revisiting the voltage-sensitive dye (VSD) RH795 for its ability to stain neurons through the ganglion sheath. We found that bath-application of RH795 specifically stains membranes of neurons and axons *without* removing the ganglion sheath. We tested various nervous systems (cricket, earthworm, and crab), and in all cases we observed that RH795 had a stronger affinity for neural membranes than to the ganglion sheath, such that the sheath itself was not strongly stained. Besides being an extraordinary anatomical marker for cell membranes, RH795 allowed us to record neuronal activity through the sheath. For this, we used the stomatogastric ganglion (STG) of the crab *Cancer borealis*, a classic model for studying neural circuit connectivity and neuromodulation on the single cell level. In the STG, the ganglion sheath is typically removed via microsurgery to enhance visibility in the ganglion and facilitating intracellular recordings. Desheathing is also necessary to allow some of the most commonly used voltage-sensitive dyes (for example ANEP dyes) to label neural membranes in the STG [13].

The STG contains the pyloric and gastric mill central pattern generators (CPGs, which control the filtering and chewing of food, respectively), and like other CPGs [14]–[20], they produce regular and predictable oscillatory activities even in isolated preparations. Both CPG circuits in the STG have been characterized in great detail [18], [21], [22] using mostly traditional extra- and intracellular electrophysiology after desheathing. The gastric mill CPG is influenced by sensory pathways, such as the proprioceptive anterior gastric receptor (AGR, [23]). Even small changes in spontaneous AGR activity cause significant changes in the activity of the gastric mill motor neurons [23]. We found that desheathing the STG caused subtle changes in neural activity, namely that rhythmic oscillations in the firing frequency of AGR can no longer be observed after the ganglion is desheathed. RH795, however, specifically stained the membranes of the pattern generating neurons in the STG *without* removing the ganglion sheath, allowing not only the visual identification of all neuronal somata in the STG, plus several axons, but also optical recording from individual pattern generating neurons whose activities are influenced by AGR. Since activities were recorded through the sheath, no mechanical stress had been imposed during desheathing and AGR's rhythmic activity persisted. Thus, bath-application of RH795 is a noteworthy tool for locating and recording many neurons simultaneously in living tissues, without the need for removing neural sheaths.

## Materials and methods

### Dissection

Adult crabs (*Cancer borealis*) were delivered from The Fresh Lobster Company (Gloucester, MA, USA) or Ocean Resources Inc. (Sedgwick, ME, USA). Invertebrate animals used in research are not subject to ethics approval at Illinois State University, and *Cancer borealis* is not a protected species. We adhered to general animal welfare considerations regarding humane care and use of animals while conducting our research. Crabs were kept in tanks with artificial sea water (salt content ∼1.025 g/cm^3^) made from artificial sea salt (Instant Ocean Sea Salt Mix, Blacksburg, VA, USA) for a maximum of 16 days. Tanks were kept at a temperature of 10–12°C and a 12-hour light-dark cycle. Before dissection, animals were anesthetized on ice for 20 minutes [10]. Adult crickets (*Gryllodes sigillatus*) were a gift from Scott Sakaluk (Illinois State University, Normal, IL) and earthworms (*Eisenia hortensis*) were bought at a local bait shop. We used isolated nervous systems to perform all of our experiments. In short, the nervous system was pinned down in a silicone elastomer-lined (ELASTOSIL RT-601, Wacker, Munich, Germany) Petri dish and continuously superfused (7–12 ml/min) with saline (10–13°C for *C. borealis*, room temperature for *G. sigillatus* and *E. hortensis*). Physiological crab saline consisted of: NaCl, 440 mM; KCl, 11 mM; MgCl_2_⋅6H_2_0, 26 mM; CaCl_2_, 13 mM; trisma base, 10 mM; maleic acid, 5 mM (pH 7.4–7.6). Insect saline consisted of: NaCl, 187 mM; KCl, 21 mM; CaCl_2_, 5.6 mM; MgCl_2_⋅6H_2_0, 4.1 mM and earthworm saline consisted of: NaCl, 103 mM; KCl, 1.6 mM; CaCl_2_, 1.4 mM; NaHCO_3_, 1.2 mM. Crabs were sacrificed on ice, and crickets and earthworms using 100% Ethanol. Both methods are recognized as acceptable under the AVMA guidelines for euthanasia of invertebrates.

### Extracellular recording

**Figure 1:**
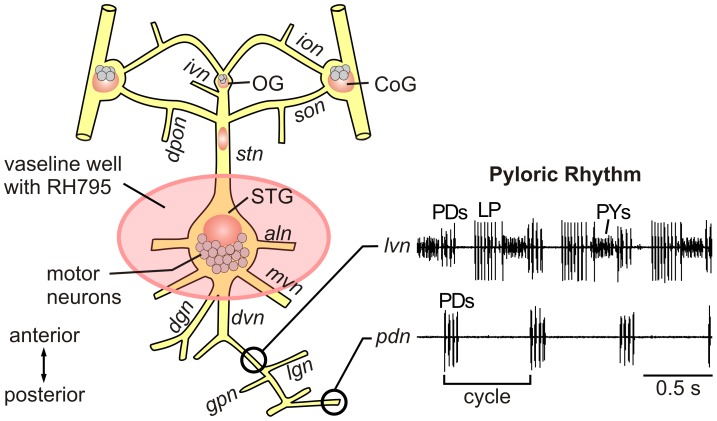
Schematic overview of the stomatogastric nervous system (STNS) of the crab, *Cancer borealis*. The pink circle marks the application site for the VSD RH795. The black circles illustrate two different extracellular recording sites and the corresponding neuronal signals. The three main neuron types (PD, LP, PY) participating in the pyloric rhythm can be monitored on the *lvn*. The *pdn* selectively shows the activity of the two PD neurons. Grey cell bodies illustrate neurons in the stomatogastric ganglion (STG), the oesophageal ganglion (OG) and the commissural ganglia (CoG). Neurons: PD, pyloric dilator neuron; LP, lateral pyloric neuron; PY, pyloric constrictor neuron. Nerves: *ivn*, inferior ventricular nerve; *ion*, inferior esophageal nerve; *son*, superior esophageal nerve; *dpon*, dorsal posterior oesophageal nerve; *stn*, stomatogastric nerve; *aln*, anterior lateral nerve; *mvn*, median ventricular nerve; *dgn*, dorsal gastric nerve; *dvn*, dorsal ventricular nerve; *lvn*, lateral ventricular nerve; *gpn*, gastro pyloric nerve; *lgn*, lateral gastric nerve; *pdn*, pyloric dilator nerve.

We used the stomatogastric nervous system (STNS) of *C. borealis* to perform activity measurements. Neuronal activity was recorded extracellularly on one of the main motor nerves. The generalized recording setup is shown in Figure 1. For monitoring the pyloric rhythm we recorded either the lateral ventricular nerve (*lvn*) or the dorsal ventricular nerve (*dvn*); for the gastric mill rhythm we recorded DG and the GMs extracellulary on the dorsal gastric nerve (*dgn*) and LG on the lateral gastric nerve (*lgn*) (Fig. 1). The gastric mill cycle period was defined as the duration between the onset of an impulse burst in LG and the onset of the subsequent LG burst. AGR activity was assessed with extracellular recordings of the *dgn*, *stn* (stomatogastric nerve) and/or *son* (superior oesophageal nerve). AGR activity was measured as instantaneous firing frequency (inst. ff.) as determined by reciprocal of the interspike interval. Mean values for all gastric mill-related parameters were determined from measurements of 20 consecutive cycles of gastric mill activity. For phase analysis, AGR inst. ff. was normalized to the minimum and maximum frequencies measured in each cycle. In some experiments the pyloric dilator nerve (*pdn*), which contains the axons of the two pyloric dilator (PD) neurons, was recorded in addition. The PD neurons are part of the pacemaker ensemble of the pyloric circuit [21]. We used petroleum jelly wells and subsequent measurements of field potential changes between two stainless steel wires (one inside and one outside of each well) to extracellularly record action potentials. The differential signal was recorded, filtered and amplified with an AC differential amplifier (A-M Systems Modell 1700, Carlsborg, WA, USA). Files were recorded, saved and analyzed using Spike 2 Software (version 7.11; CED, Cambridge, UK).

### Stimulation parameters

To show the rhythmic modulation of the AGR inst. ff. we elicited a gastric mill rhythm. For this, the ventral cardiac neurons (VCNs) were activated by stimulating the dorsal posterior oesophageal nerve (*dpon*) extracellularly with 10 consecutive stimulus trains of 15 Hz stimulation frequency, 6 s train durations and 4 s intertrain intervals [24].

### Preparation and application of the dyes

In all experiments we used the styryl dye RH795 (Pyridinium, 4-[4-[4-(diethylamino)phenyl]-1,3-butadienyl]-1-[2-hydroxy-3-[(2-hydroxyethyl)dimethylammonio]propyl]-,dibromide/172807-13-5; Biotium, Hayward, CA) which was first synthesized by Rina Hildesheim and Amiram Grinvald [25]. A 10 mM stock solution was prepared by diluting 5 mg dye in 854 µl of ultrapure water and kept in darkness at 4°C. Immediately before bath-application, the stock solution was diluted in saline to the final concentrations of 0.3 mM. Figure 1 shows the application setup for the STG. Application procedures were similar for cricket and earthworm ganglia. In contrast to most other studies, we did not desheath the STG for performing the optical recordings. Rather, a petroleum jelly well that isolated the STG from other parts of the STNS was built and approximately 50 µl of RH795 were bath-applied to the well (similar to [26]). The part of the STNS that was located on the outside of the well was constantly superfused with chilled saline (10–12°C) during dye application. The dye was applied for 30–60 minutes, after which the petroleum jelly well was removed and the whole preparation was superfused with chilled saline for the remainder of the experiment. For comparing the visibility between stained/non-desheathed and stained/desheathed ganglia, we desheathed the STG and took a count of the visible cells in some experiments.

### Optical imaging and picture processing

For comparing the fluorescence before and after desheathing, we used a 5 mega pixel color CMOS camera (TCA-5.0C, Ample Scientific LLC, Norcross, GA, USA) and TSView software (Version 7.3.1.7, Tucsen Imaging Technology Co., Fujian, China). Fluorescent excitation light was provided by a CoolLED system (narrowband LED with 535 nm; Yorktown Heights, NY) and fluorescence emission was detected using a 560 nm beam splitter and a 570–640 nm emission filter (Olympus, Center Valley, PA). Excitation light intensities and imaging exposure time varied and were adjusted to the individual preparation. We either used a 20× objective (XLUMPlanFL N, NA 1.0, WD 2.0 mm, cc = water; Olympus Corporation, Tokyo, Japan) or a 10× objective (UMPlanFL N, NA 0.30, WD 3.3 mm, cc = water; Olympus Corporation, Tokyo, Japan) mounted on an upright epifluorescence microscope (modified BX51, Scientifica, East Sussex, UK). For recording fluorescence changes (‘optical imaging’) the MiCam02 imaging system and software (Brain-Vision Analyzer, BV-ANA, Version 11.08.20; SciMedia Ltd, Tokyo, Japan) were used with the HR (High Resolution) camera (6.4×4.8 mm actual sensor size) set at either 384×256 pixel spatial resolution for high resolution photos or at 192×128, 96×64, or 48×32 pixel spatial resolution for optical imaging. A temporal resolution of 2–20 ms was chosen. Typical recordings lasted between 16 and 32 seconds, and were repeated many times in a given experiment.

### Data Analysis

Averaging of signals was performed using associated scripts for Spike2 (www.neurobiologie.de/spike2). The cycle period of the pyloric rhythm was defined as the duration between the onset of a PD neuron burst and the onset of the subsequent PD burst. In some experiments, cycle-based and spike-triggered averaging were used to improve signal quality, according to the protocols given in [13] and analyzed in Spike2. Cell counts were performed in dark-field illumination using a Wild M8 stereomicroscope (Heerbrugg, Switzerland) or an upright epifluorescence microscope (modified BX51, Scientifica, East Sussex, UK), before and after desheathing and before and after staining with RH795.

In selected experiments, the frequency components of optical and extracellular signals were plotted as a spectrogram. In these cases, frequency analysis was restricted to a frequency band of 0–10 Hz, which contains the main frequencies present during the pyloric and gastric mill rhythms. Additionally, the correlation of the frequency distribution was calculated for each point in time during the recordings, allowing the direct comparison of the frequency components of different neurons. This allows for a quantification of the correlative strength of activity from neuron-to-neuron, along with showing if a given neuron participates in a particular rhythm at all times. Time resolution was 0.1 seconds and frequency resolution was 0.1 Hz (resulting in 100 frequency steps from 0–10 Hz that were correlated at each time point).

Waveform correlations were calculated by multiplying two waveforms together, point by point, and summing the products. The sum was normalized to allow for waveform amplitudes and the number of points. The reference waveform was then repeated for all time bins. Results range between +1.0, meaning the waves are identical except for amplitude through 0 (uncorrelated) to −1.0, meaning identical but inverted. The bin width corresponded to the sampling bin width.

Optically recorded neurons were identified after cycle-based averaging by comparing waveform shape, phasing, and timing of action potential occurrence relative to extracellular recordings (similar to the identification of intracellularly recorded neurons).

### Statistics and figure making

For spreadsheet analysis, Excel (version 2010 for Windows, Microsoft) or SigmaPlot (version 11 for Windows, Systat Software GmbH, Erkrath, Germany) were used. Normally distributed data are given as mean ± SD. “N” denotes the number of animals, while “n” is the number of trials. In all figures significance is indicated using * (p<0.05), ** (p<0.01), *** (p<0.001). Statistical tests for data analysis were t-test, Pearson product-moment correlation coefficients or One Way ANOVA with Holm Sidak posthoc test. Final figures were prepared with CorelDraw (version X3 for Windows, Corel Corporation, Ottawa, ON, Canada).

## Results

One of the major advantages of small system approaches in neuroscience is the use of identified neurons or neuronal populations [27]–[29]. However, recordings are often hindered by **(a)** the difficulty to localize neuron somata and arborizations in living tissue and **(b)** having to penetrate neurons through non-neural tissue such as the ganglion sheath in most invertebrate preparations. Here, we revisit RH795, a voltage-sensitive dye used for optical imaging, and assesses its ability **(a)** to stain populations of neurons in living tissue and **(b)** to allow recording through the ganglion sheath. We used three different model systems with partly identified neural circuits to test RH795: the cricket metathoracic ganglion, the earthworm ventral ganglion chain, and the crustacean stomatogastric nervous system.

### RH795 is a good anatomical marker for cell body location

**Figure 2:**
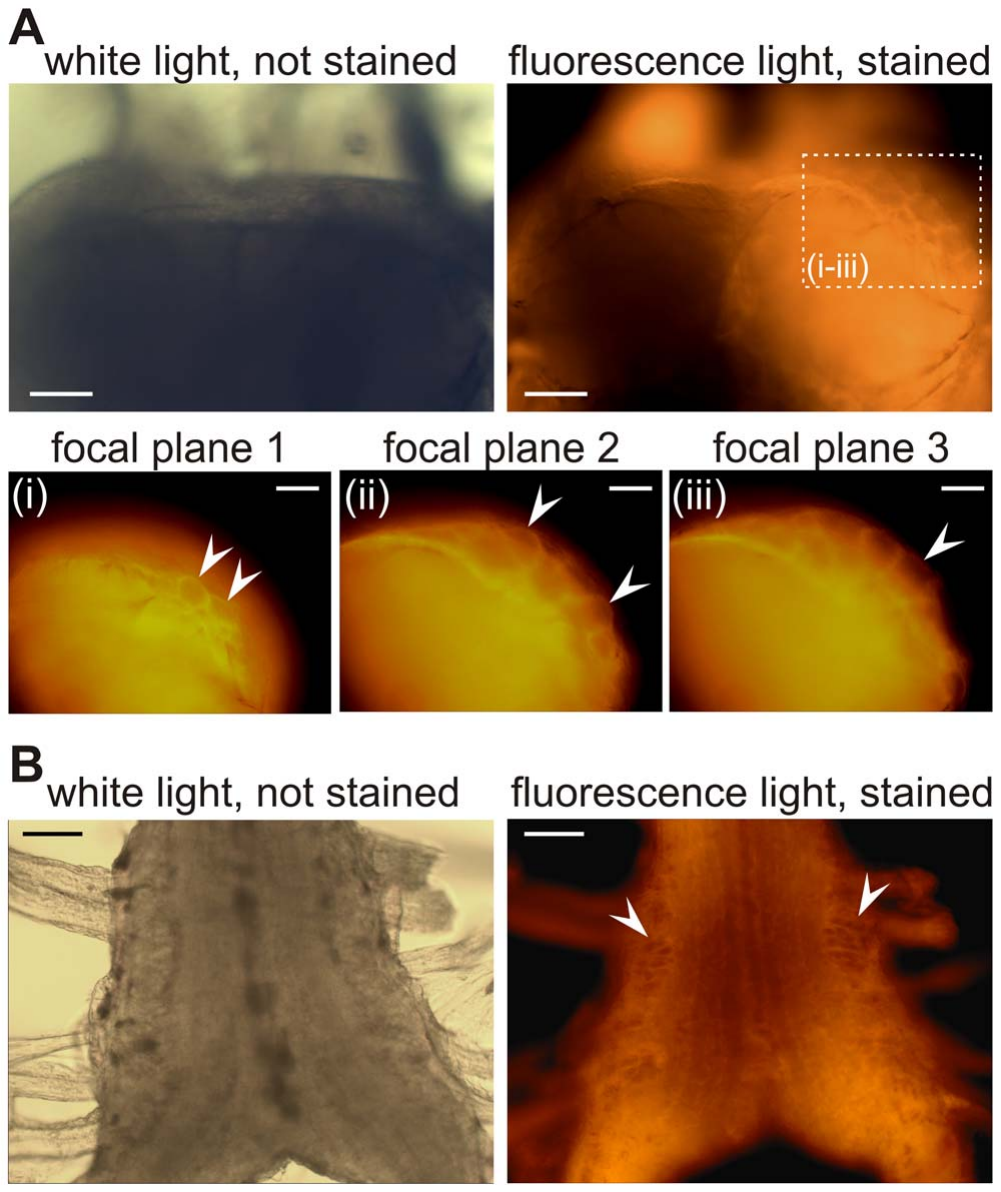
RH795 stains through the ganglion sheath and allows the visualization of cell body location. **A:** Metathoracic ganglion of the cricket *G. sigillatus* before (white light) and after staining (fluorescence light). Scale bar is 100 µm. (**i–iii**) Higher magnification of the dotted area in the picture above. Changing the focal plane from dorsal (i) to ventral (iii) revealed multiple layers of distinct neurons (see arrows). Scale bar is 50 µm. **B:** Subpharyngeal ganglion of the earthworm *E. hortensis* before and after staining with RH795. Large neuronal somata were visible after staining (see arrows). Scale bar is 100 µm.

After dissection, the isolated nervous system was placed into a Petri-dish containing physiological saline. For the control, the ganglion was photographed in white light before RH795 was bath-applied. RH795 was left on for one hour and subsequently washed out with saline. Fluorescence imaging began 15 minutes after the washout was started. We found similar results for all ganglia used: before dye application, very few, if any, neuronal somata or arborizations were visible. Figure 2A shows a cricket metathoracic ganglion before and after dye application. As in most insect thoracic ganglia, the cricket ganglion has distinct areas for neuropil structures and cell bodies, respectively [30]. This broad distinction was visible before staining: lighter areas around the outline of the ganglion, where the cell bodies are located, and denser neuropil areas in the middle of the ganglion. However, no individual neural structures could be discerned. After dye application, the outlines of many neuronal somata were visible in the fluorescent light. Moving the focal plane of the microscope from dorsal to ventral revealed multiple layers of neurons that were all individually identifiable (arrows in Figs. 2A(i)–(iii)).

We found similar results for the staining of the earthworm ventral ganglia (Fig. 2B): before dye application, very few individual cell bodies were visible. After staining with RH795 the outlines of distinct neuronal somata were evident, and in particular at the lateral borders, where large neuronal somata are located [31]. As for the cricket ganglion, changing the focal plane revealed the location of different distinct neuronal layers.

**Figure 3:**
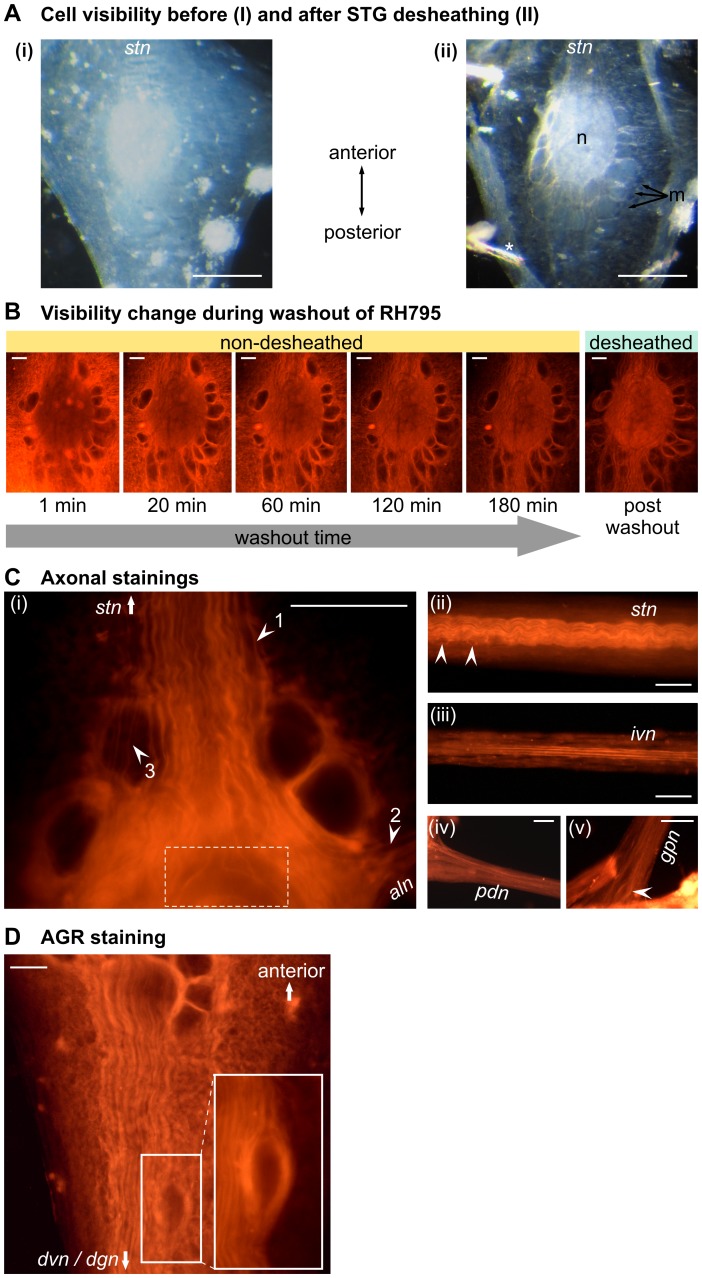
RH795 staining in the stomatogastric nervous system of the crab, *Cancer borealis*. **A:** Cell visibility before (**i**) and after desheathing (**ii**) the STG (note that the ganglion was not stained here). Pictures show the same ganglion, illuminated with a dark-field condenser and taken using the same light intensity, magnification, and camera settings. n, neuropil; m, motor neurons; * minuten pin. Scale bar is 100 µm. **B:** RH795 stains STG neurons through the ganglion sheath and causes a clear and long-lasting staining of neural structures. Comparison of visibility of the non-desheathed ganglion during 180 min of washout of the dye (left 5 pictures) and after desheathing (right). Immediately after removing the dye, the sheath still contained a lot of dye (1 min), but contrast and visibility improved quickly. The dye caused a long-lasting staining through the ganglion sheath without excessive residual staining of the sheath. Visibility did not further improve after desheathing the ganglion. Scale bar is 50 µm. **C:** RH795 revealed fine neuronal structures like neurites, axons and varicosities. (**i**) Anterior part of the STG showing individual axons entering the ganglion via the *stn* (see arrows). Distinct neurites in the STG neuropil could be identified, which is impossible with regular light microscopy. (**ii**) Middle part of the non-desheathed *stn*. Individual axons and varicosities (arrows) were visible after staining. (**iii**) Staining of the *ivn*, (**iv**) the *pdn* and (**v**) the *gpn*. Arrow indicates the location of the soma of the gastro-pyloric receptor neuron GPR. Scale bar is 100 µm. **D:** The AGR soma shown here stained with RH795 is anatomically isolated from other STG neurons, located more posteriorly than the somata of STG motor neurons. Scale bar is 50 µm.

In contrast to most other arthropod ganglia, the STG of the crab, *Cancer borealis*, contains only 26 neurons, all of which are individually identifiable by their firing and axonal projection pattern [21]. Due to the extensive knowledge about the circuit connectivity and the neural activity, we used the STG to study RH795 in more detail. In none of the crustacean species studied are the location of STG neurons fixed, i.e. neuron location differs from animal to animal [32]. Hence, the desheathed ganglion is typically used to increase visibility in the ganglion and to facilitate access to the neurons. Indeed, desheathing improved visibility in our experiments as well (Fig. 3A): On average, the location of 10.3±2.9 somata (N = 15) could be determined before desheathing. After desheathing the number of unambiguously identifiable neurons increased significantly to 19.8±3.2 (N = 55, p<0.001; One Way ANOVA with Holm Sidak posthoc test). Voltage-sensitive dyes have been used to study small invertebrate nervous systems for some decades (primarily absorption VSDs [33], [34]). We have previously shown that fluorescent VSDs, such as ANEP dyes [35], [36] and RH795 [26], stain neural membranes in the STG and allow the visual recognition of most STG neurons [13], [26]. However, VSDs are typically applied after removing the ganglion sheath [2], [4]–[6], [9], [11], [35], [37], [38]. Here, as for the cricket and earthworm, we bath-applied RH795 to the non-desheathed ganglion to test its ability as a neural marker without removing the ganglion sheath. RH795 was bath-applied for 60 minutes, after which dye washout was started and another cell count was taken. At this point in time, the sheath still contained large amounts of dye, obscuring some of the fine details of the intra-ganglionic structures (Fig. 3B, left). Nevertheless, most of the 26 STG cell bodies could be located (21.2±3.6, N = 21, significantly different from before desheathing/not stained, p<0.01; One Way ANOVA with Holm Sidak posthoc test). Contrast and visibility improved within the next 20 minutes, after which a stable staining was observed for at least the following hour and up to 3 hours (Fig. 3B). Apparently, the dye had stained through the ganglion sheath without excessive residual staining of the sheath. To confirm that indeed neuronal membranes had been stained, we desheathed the ganglion after the staining procedure and compared the visibility before and after desheathing (Fig. 3B, right). There were no obvious differences in location, size and shape of the neurons, and the number of neurons that could be accounted for by visual inspection of the ganglion was not significantly different from the non-desheathed/stained ganglia (19.5±4.8; N = 6; p>0.2; One Way ANOVA with Holm Sidak posthoc test).

### RH795 reveals fine neuronal structures like neurites, axons and varicosities

RH795 also revealed individual axons in the STG (Fig. 3C (i)), which typically cannot be seen without fluorescence staining even when the ganglion is desheathed (for comparison see Fig. 3A (ii)). Figure 3C (i) shows the anterior part of the STG, where the *stn* enters the STG. The *stn* connects the STG to three other ganglia as well as to the rest of the CNS and contains approximately 60 axons [39]. A striking feature is that axons that enter the STG tend to be larger in diameter and more spread out than they are in middle parts of the same nerve [39]. For example, axons cover over a width of ∼200 µm when they enter the STG, while they are bundled and stacked and take up less than 100 µm in the middle of the *stn*. After staining with RH795 *stn* axons were clearly visible, both in the STG and in the *stn*. In some cases, we could even identify specific axons: Figure 3C(i) shows an axon entering the STG via the *stn* and leaving the STG (arrows 1 and 2) via one of the side nerves (*aln*). This is the axon of the CD1 neuron (cardiac sac dilator neuron 1), the only neuron that projects an axon via the *stn* to the *aln* [40], [41]. RH795 also revealed structures usually invisible even after desheathing, such as axons lying on top of neurons (arrow 3) and the shape of primary neurites (dotted square). We tested whether individual axons could also be traced in nerves with bundled axons by bath-applying RH795 to individual nerves. We compared nerves containing densely bundled axons (middle part of the *stn*) with motor nerves containing only a few axons (*pdn*) and sensory nerves (*gpn*: gastro pyloric nerve [42] and *ivn*: inferior ventricular nerve [43]). We used the same procedure as for the ganglion staining. In general, we found that RH795 clearly stained axon bundles without strong residual staining of the nerve sheath. Staining the middle part of the *stn* revealed many individual axons and the staining was good enough to trace a particular axon over a large distance (Fig. 3C(ii)). In addition, several varicosities could be seen in the vicinity of the *stn* axons (arrows in Fig. 3C(ii)). Identifying individual axons was easier in nerves containing only a few axons, such as the *ivn*. The *ivn* contains only 8 axons [44], namely those of two identified projection neurons and 6 axons of so far undescribed neurons. After staining with RH795 we could clearly separate 6 of the 8 axons from each other (Fig. 3C(iii)), even in a single optical focal plane. Stainings of the *pdn* (Fig. 3C(iv)), through which the two PD neurons project to the pyloric muscles, revealed two large axons plus at least one small diameter axon (possibly sensory). In the case of the *gpn* (Fig. 3C(v)), the dye revealed not only individual axons, but also the location of the soma of the gastro-pyloric receptor neuron [45], a muscle stretch receptor, which so far has only been located with retrograde backfill stainings in fixed tissues [42]. Finally, RH795 allowed us to visualize the soma of the sensory neuron AGR [46] with relative ease (Fig. 3D). The AGR soma is located in the posterior part of the STG, separated from the motor neurons. The AGR soma is embedded in in connective tissue and can typically not be detected without desheathing.

In summary, RH795 staining allowed the comprehensive mapping of structural details never achieved in white light or dark-field views of intact STNS nerve tissue, demonstrating its ability to function as an anatomical marker.

### Desheathing the STG causes subtle changes in neuronal activity

**Figure 4:**
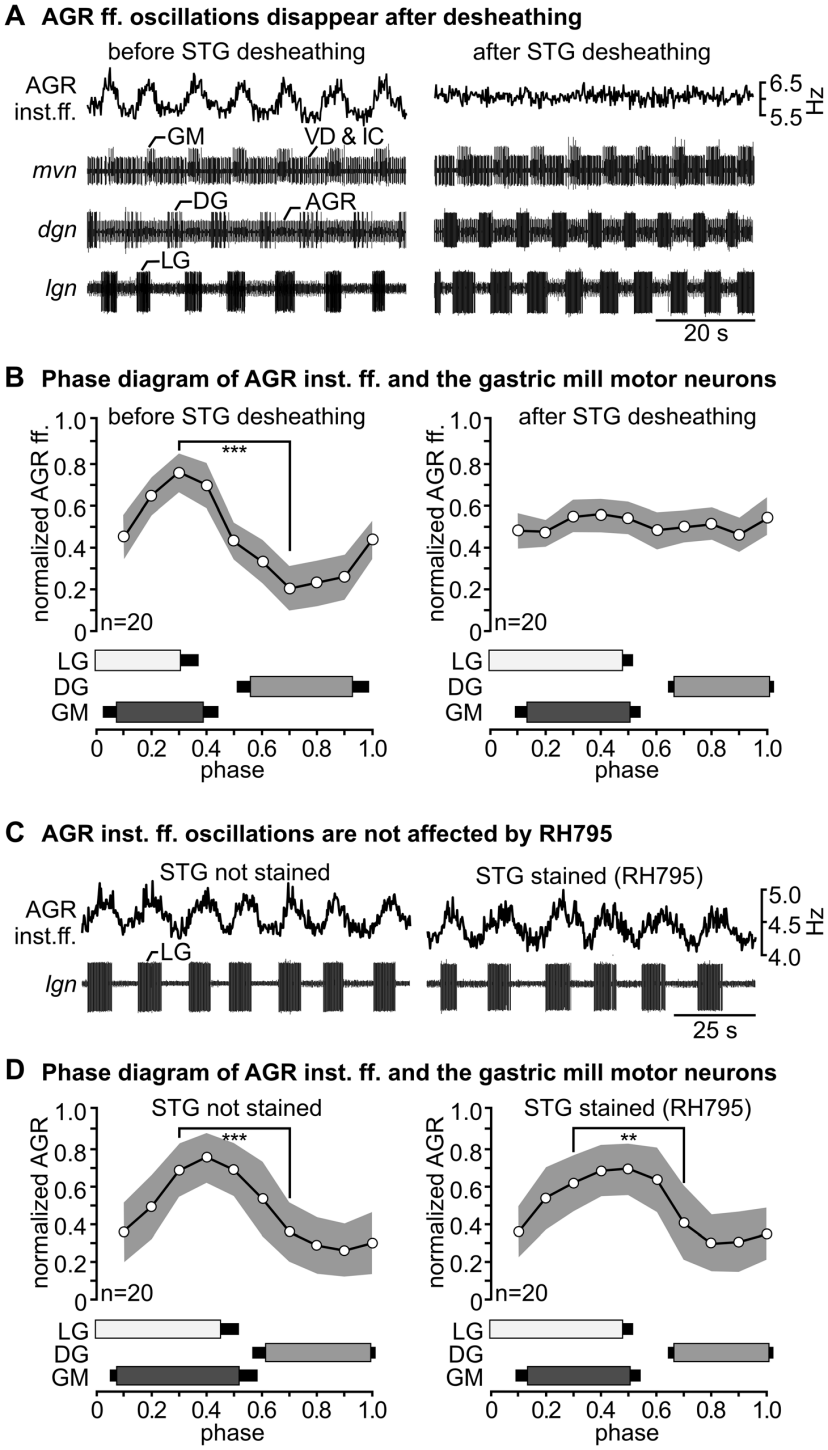
Desheathing causes subtle changes in neuronal activity. **A:** Rhythmic changes in AGR inst. ff. disappear after desheathing. During a gastric mill rhythm, AGR inst. ff. (top trace) changed rhythmically. Bottom traces: extracellular recordings of *mvn*, *dgn* and *lgn* showing the timing of the gastric mill rhythm. Left: before desheathing; right: after desheathing the STG. Raw data are included in Raw Data.xlsx S1. **B:** Phase diagram of normalized averaged AGR inst. ff. and the gastric mill motor neurons LG, GM and DG before (left) and after (right) desheathing, from the same animal. AGR inst. ff. was binned (bin width 0.1). The grey surface shows the standard deviation. ***significant difference with p<0.001. **C:** Rhythmic changes in AGR inst. ff. were not affected by RH795. Left: non-desheathed STG, before staining, right: non-desheathed STG after 1 h staining and 20 minutes washout. **D:** Phase diagram of the same animal before (left) and after (right) staining with RH795. For detail see (B). Phases 0.3 and 0.7 were significantly different. ***p<0.001 before staining and **p<0.01 after staining. There was no significant difference between the mean values of all phases before and after staining (see text for details).

The extraordinary access to neurons in the STG also allows testing the ability of RH795 for measuring membrane potential changes. The main reason for removing the ganglion sheath of the STG is that desheathing facilitates the recording of membrane potential changes with glass microelectrodes. Generally, the pyloric and gastric mill motor patterns are similar after desheathing to those generated without desheathing and to those generated *in vivo* [44], [47]. However, quantitative comparisons are absent. It is understood that neuromodulators are released from descending neurons in the STG [21], [27], and that extracellular peptidases limit the actions of these molecules and significantly affect neuronal output [48]. Desheathing may compromise this balanced system of peptidase activity and diffusion of neuromodulators and lead to changes in the response of neurons when neuromodulators are released. The anterior gastric receptor (AGR) neuron shows a conspicuous absence of response to neuromodulatory input, for example. AGR possesses neurites with postsynaptic structures in the STG neuropil [49], but reports about influences of STG motor activity and neuromodulator release on AGR are absent. Previous studies have failed to show any influence of pyloric, gastric mill, or descending neurons on the activity of AGR [49]. We hypothesized that this conspicuous absence is due to the missing ganglion sheath in these experiments. Hence, we tested whether the presence of a sheath was influencing the activity of AGR, and found that desheathing can have subtle, but consistent influences on its firing patterns. Figure 4A shows an extracellular recording of the activity of the sensory neuron AGR [23] during a gastric mill rhythm elicited by stimulation of the sensory nerve *dpon* ([24], for details see Materials and Methods). AGR activity showed small fluctuations in firing frequency *without* desheathing that were timed with the gastric mill motor neurons (Fig. 4B). On average, AGR instantaneous firing frequency (inst. ff.) was significantly higher during the activity phase of the lateral gastric motor neuron (LG; AGR inst. ff. 6.29±0.27 Hz, n = 20) than during its functional antagonist, the dorsal gastric neuron (DG; AGR inst. ff. 5.88±0.22 Hz, n = 20; t-test, p<0.001, n = 20). When we compared the change of AGR inst. ff. at different phases of the gastric mill rhythm, we found that the maximum occurred at phase 0.3 and the minimum at phase 0.7, and that AGR inst. ff. was significantly different between these phases (t-test, p<0.001, n = 20). After desheathing, however, AGR activity was tonic and no longer timed with the gastric mill rhythm (Figs. 4A & B, right). No significant differences were present between phases 0.3 and 0.7 (t-test, n = 20, p>0.6). In all animals tested (N>10), desheathing abolished the oscillations in the AGR firing frequency.

### RH795 does not alter neuronal activity in the STG

We were curious whether RH795 would not only allow us to stain all neurons in the STG, but also would enable us to record membrane potential changes in the stained tissue using optical imaging without desheathing. As a prerequisite, we tested whether application of RH795 interfered with neuronal activity patterns. This was not the case, as also reported previously [26]: pyloric and gastric mill motor patterns could still be observed after staining the ganglion. Figure 4B shows that even the rhythmic changes in AGR inst. ff. where still present after RH795. In both conditions, non-stained and stained (but non-desheathed), AGR's inst. ff. was significantly higher (t-test, p<0.001, n = 20) during the activity phase of LG (mean ff. 4.93±0.24 Hz, n = 20) than during the DG phase (Fig. 4D, mean ff. 4.13±0.19 Hz, n = 20). We also found a significant difference between phases 0.3 and 0.7 for both conditions (t-tests, p<0.001 STG non-stained; p<0.01 STG stained, n = 20), which is consistent with our previous observations. In fact, there was no indication that staining the STG with RH795 had any influence on the AGR activity. A comparison of the mean values of all phases before and after staining shows no statistical difference (t-test, p>0.8, n = 20 for each comparison). In summary, the gastric mill-timed oscillations in the AGR firing frequency were, unlike after desheathing (Fig. 4A), still present after staining with RH795 (Fig. 4C & D), indicating that removing the ganglion sheath can in some instances change neuronal activity. The use of RH795 may circumvent these issues since it allows localizing the AGR soma (Fig. 3D) without influencing AGR activity, and hence may allow AGR activity measurements without desheathing.

### Neuronal activity can be imaged through the ganglion sheath

The neurons in the pyloric circuit in the STG are well-described [21], and their membrane potential oscillations and firing phases relative to other neurons and extracellularly recorded activity patterns are sufficient to unambiguously identify most neuron types participating in the gastric mill and pyloric rhythms [21]. The pyloric rhythm is triphasic, has a cycle period of 0.5–2 seconds and can be monitored by recording the activities of the lateral pyloric (LP), pyloric dilator (PD) and pyloric constrictor (PY) neurons on the corresponding motor nerves (*dvn* or *lvn* for monitoring all three neuron types, *pdn and pyn* for exclusively monitoring the PDs and PYs, respectfully).

**Figure 5:**
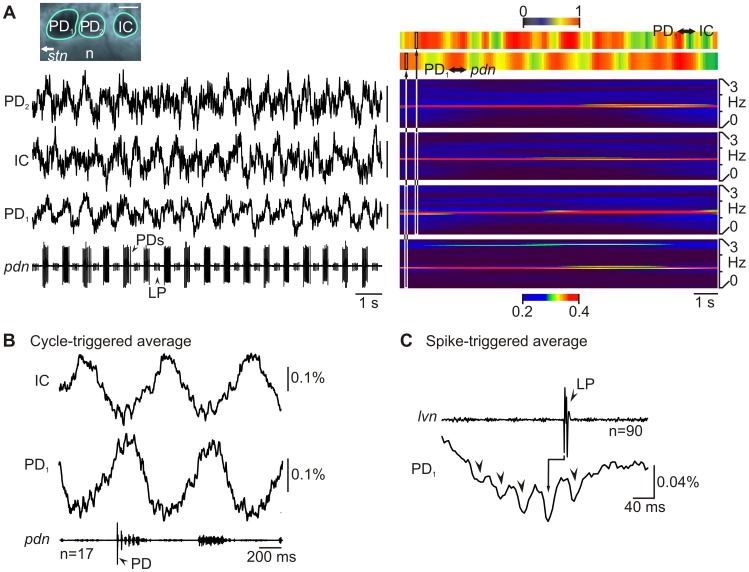
Optical imaging through the ganglion sheath. **A:** Left, Single-sweep simultaneous optical recording of IC and the two PD neurons, along with an extracellular recording of the *pdn*. In this particular recording the *pdn* also shows the action potentials of LP. Top: Photo showing the three somata selected for recording. Scale bar is 50 µm; n, neuropil. PDs and IC showed rhythmic and alternating changes in their fluorescence. Vertical scale bars are 0.04%. Right: spectrogram showing the major frequency components of optical and extracellular recordings. Warmer colors indicate higher power. Top traces, correlation of frequency distribution (1–10 Hz) for each time point for PD and *pdn*, as well as for PD and IC. There was a high correlation of the frequency components at all points in time. Note the different time scale (slightly compressed to fit page). **B:** Cycle-triggered average of PD and IC, plus *pdn*, showing the phase dependence of the optical signal. The first PD spike in each cycle was used for triggering. **C:** LP spike-triggered average of *lvn* and PD optical recording showing LP-timed synaptic inhibition in PD and its temporal dynamics.

To test whether RH795 permits optical recording of STG neuron activity through the ganglion sheath, we monitored the changes in fluorescence over time and compared them to the extracellularly recorded pyloric motor pattern *without* desheathing the STG. Figure 5A shows a high-resolution photo of three adjacent STG neurons (20× objective) and the corresponding optical recordings from three selected regions of interest. The corresponding extracellular recording of the *pdn* (bottom trace) shows the timing of the pyloric rhythm. All recordings were taken simultaneously (non-averaged data). While no consistent change in fluorescence was detected outside of neural structures (data not shown), the three neuronal somata showed fluorescence signals that correlated with the phasing of the pyloric motor neurons on the *pdn* (for visualization, see movie S1). Previously published measurements of the desheathed ganglion demonstrate that these changes in fluorescence represent the slow membrane potential oscillations of these neurons [13]. Often, we were able to identify neurons solely based on their single-sweep optical signal (without averaging over multiple cycles) because the signal-to-noise ratio was high enough for a distinct comparison with the extracellular recording. For example, the top and bottom traces in Figure 5A show a clear increase in fluorescence when the PD neurons were active, while the middle trace shows antiphasic activity that was in time with the LP neuron. Further analysis (cycle-triggered average, Fig. 5B) revealed that the two PD-timed optical recordings indeed were PD neurons and that the LP-timed optical recording was the inferior cardiac (IC) neuron, which is active at approximately the same phase of the pyloric cycle as LP [21]. For averaging, we used the simultaneously recorded activity of the PD neurons on the *pdn* to determine the beginning and end of pyloric cycles, and then averaged across several pyloric cycles (see also [13]). Since averaging relies on the rhythmic activity of the motor neurons, this procedure facilitated neuron identification, and also revealed more of the neuronal subthreshold membrane potential changes, in particular of the PD neuron recording (since its action potentials were used to time the average). When we compared the frequency of the pyloric rhythm measured from the optical signal with that measured from the spike activity on the *pdn*, we found that they were virtually identical: Comparing the spectrograms of extracellular recording and optical signal (see Materials and Methods) revealed a high correlation in the frequency band that corresponded to the pyloric cycle frequency (Fig. 5A, right) and there was a high correlation between the frequency components of the optical signals and the extracellular recording (top heat maps). All recordings showed a significant correlation (p<0.05, n = 131 for all comparisons) in the frequency domain. The highest correlation was found between the two PD neurons (Pearson product-moment correlation coefficients: IC and PD_1_: 0.83±0.13, PD_2_ and IC: 0.88±0.1, PD_1_ and PD_2_: 0.91±0.09, IC and *pdn*: 0.65±0.1, PD_1_ and *pdn*: 0.68±0.07, PD_2_ to *pdn*: 0.61±0.12).

No individual action potentials could be detected in single-sweep recordings - a possible consequence of the slow sampling intervals of the camera (4 ms) and an undersampling of the fast membrane potential changes during the action potential. However, when we used spike-triggered averaging (a method typically used for identifying neurons in this system during intracellular recordings [50]), we were able to measure synaptic interactions between neurons. PD receives inhibitory synaptic input from LP [21], and we have shown previously that synaptic potentials can be detected using optical recordings after spike-triggered averaging [13], [37], [38]. Here, we triggered on the LP action potentials on the extracellular *lvn* recording and simultaneously recorded PD fluorescence emission. Distinct IPSPs were obvious in the averaged signal of PD that occurred in response to the LP action potentials (Fig. 5C), indicating that VSD imaging of synaptic events through the sheath is feasible and effective. The signal-to-noise ratio after averaging was high enough to also recognize the temporal synaptic dynamics of the LP-PD synapse. When we compared the averaged fluorescence change in PD that was elicited by the first LP action potential in the LP burst to that elicited by the last action potential, a clear decrease was obvious. This is due to synaptic depression present at this synapse [51].

**Figure 6:**
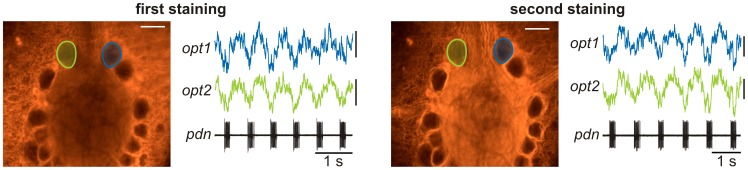
Repetitive staining permits long-term experiments. Photos with regions of interest and simultaneous single-sweep recording of two pyloric neurons, plus *pdn*. Left: first staining; right: second staining. Recordings were taken roughly 5 hours apart from one another. Staining duration was 1 hour in each case. Scale bars: 100 µm (photo) and 0.1% (optical recordings).

For all optical recordings, we recognized a slow decrease in fluorescence intensity over time along with a diminution of the signal-to-noise ratio. Whether the reduction in fluorescence and signal quality was caused by bleaching or a slow washout of the dyes is unclear, but repetitive application of the dye was able to re-establish the staining and the signal-to-noise ratio (Fig. 6). Continuous application thus allowed long-lasting staining experiments.

### Simultaneous recordings of multiple gastric mill motor neurons through the ganglion sheath

Because neurons in the STG are rather large (10–100 µm in diameter) and typically arranged in a single layer, they are easily accessible for intracellular recordings if the ganglion is desheathed. However, as we have shown above, desheathing can cause subtle changes in neural activities. RH795 might solve this dilemma by allowing us to localize and record most STG neurons simultaneously without the need for desheathing. The counterpoint to this would emphasize that the spike activity of most STG neurons can be recorded extracellularly. However, synaptic potentials and membrane potential changes cannot be observed. Also, not all STG neurons can be identified uniquely using extracellular recordings: one example is the two PD neurons shown in Fig. 5A. There is no single nerve that contains individual PD axons for a separation of their activities. Yet, the PD neurons are strongly electrically coupled and usually they act in unison. For other neurons this is not clear. The 4 GM (gastric mill) neurons, for example, are weakly electrically coupled, but are active together since they receive similar synaptic input [52]. Since their spikes on extracellular recordings are small and intermingled, they cannot reliably be separated on such recordings. As is the case for the PD neurons, there is no nerve that contains individual GM axons. In contrast to PD neurons, however, there are indications that the 4 GM neurons have dissimilarities: The average number of GM axons on the *mvn* is 2.95 [53] (and not 4), indicating that in any given animal, only a subset of GM neurons project through a given nerve and that, consequently, the GM neurons do not necessarily share the same axonal projection pathways. Here, we were using RH795 to simultaneously record the 4 GM neurons to determine if they were active in unison, and if not, how conspicuous their differences were.

**Figure 7:**
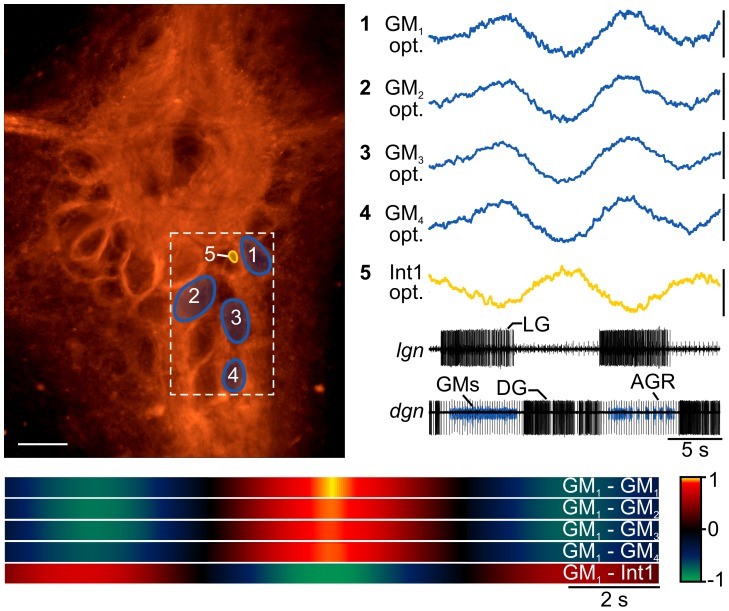
RH795 allows the simultaneous recording of gastric mill neurons through the ganglion sheath. Left: photo of STG with 5 regions of interest representing the locations of the 4 GM neurons and Int1. Actual optical recording region indicated by dotted-white lines. Scale bar is 100 µm. Right: simultaneous optical recording of GMs and Int1. Scale bars are 0.4%. Bottom traces: extracellular recordings of *lgn* and *dgn*, showing the burst activities of LG, DG and GMs. Note that GM spikes (blue) are small, preventing their individual identification on the extracellular recording. Bottom: Waveform correlation of GMs and Int1, showing high synchrony of the 4 GMs and antiphasic correlation of GMs and Int1. Colors code correlation factor (1: strong correlation; 0: no correlation; −1: strong antiphasic correlation). GM_1_ was used as a reference for all correlations, with the top trace showing the GM_1_ autocorrelation.

Figure 7 shows a photo of the STG taken with the 10× objective after RH795 staining, and the corresponding optical recordings from 5 selected regions of interest. The 4 blue traces show single-sweep recordings of the 4 GMs. The yellow trace shows an optical recording of Interneuron 1 (Int1).Int1 is one of the smallest neurons in the STG and cannot be identified on extracellular recordings, since its action potential is buried among many others on the *stn*. Due to its small soma (∼5–10 µm diameter), it also cannot be seen through the ganglion sheath, and even after desheathing, impalements are difficult. However, after RH795 staining, even small cell bodies such as that of Int1 were clearly visible without desheathing, and could be recorded optically. For comparing GM and Int1 activities, a gastric mill rhythm was elicited with *dpon* stimulation (see Materials and Methods). The corresponding extracellular recording of the *lgn* and *dgn* (bottom 2 traces) show the timing of the gastric mill rhythm after stimulation. All recordings were taken simultaneously with 20 ms sampling rate. The membrane potential changes of all 4 GM neurons, plus those of Int1 can easily be seen (visualized in movie S2). The GMs were active during the phase of the LG neuron, while Int1 was antiphasic to it, as previously described [54]. The correlation diagram (Fig. 7, bottom) shows high synchrony between the 4 GM neurons, and antiphasic correlation between GMs and Int1. As indicated by the heat maps, while all maximum GM correlation factors were >0.9, slight variations existed when comparing each GM neuron in this condition. To confirm the identity of the neurons, after imaging we desheathed the ganglion and impaled the neurons with sharp microelectrodes and compared their spike activity and membrane potential oscillations to previously published data [54], [55].

Our experiments thus show that using RH795, we can record and separate the activities of STG neurons that are inaccessible for other types of recordings without desheathing the ganglion. With respect to the 4 GM neurons, within the limitation of the used imaging technique (frame rate and signal-to-noise ratio), we found a high synchrony amongst the GM neurons.

## Discussion

We demonstrate that when bath-applied to neural tissue, RH795 permeates neural sheaths and stains sub-sheath neuronal compartments, indicating its potential as an anatomical marker without desheathing. In addition, we successfully recorded neural activity through the sheath via changes in fluorescence of neuronal membrane-bound RH795. These findings suggest the use of optical imaging with RH795 as an alternative to protocols that require the removal of the sheath prior to experimentation.

### RH795 as an anatomical marker for living neural tissue

Discriminating between individual neurons is essential in any attempt to study neural networks and their function—a task particularly difficult in living tissue and during the recording of neural activities. Most recent studies thus now comprise combinations of several techniques that allow staining the neural tissue and the simultaneous measurement of activity [56], [57]. Identifying cellular structures such as somata and axons is particularly important in light of current efforts to decipher the connectome of neural networks in many systems [58]–[60]. In the simplest case, activity measurements are performed without detailed visual guidance (‘blind recordings’) and only afterwards, neural structures are then identified (using retrograde labeling, chemical precipitation or stereotactic placement of the electrodes, for example). For single cell recording with microelectrodes, intracellular injection of (fluorescent) dyes can label the recorded neurons during the experiment, and allows the analysis of the associated neuritic arborizations after the experiments, producing an anatomical layout of the recorded cells [61]. In any of these cases, the success of the experiment usually depends on the experience of the experimenter in localizing the neurons in question. More recently, the use of fluorescence techniques has become an aid in determining the anatomical structure of otherwise unidentifiable neuronal features [62], [63]. While retrograde backfilling of axons with fluorescent dyes allows the staining of distinct neurons even before an experiment, the expression of fluorescent proteins such as GFP, calcium- or voltage-indicators in selected neural tissues has facilitated neuronal identification in living tissue [1], [64]–[66]. Targeted expression of fluorescent proteins now provides a tool for guiding electrode placement and anatomical descriptions of distinct neural tissue even in large brains. Since expression is targeted to single neuron types, several driver lines are needed to dissect whole circuits, as these consist of many neuron types. Targeted expression is thus equivalent to the identification of individual neurons in smaller nervous systems. Using Brainbow, a protein translation of fluorescent protein variants to yield a vast array of coloring, even individual cells of the same type can be visually separated [67]–[69], albeit with the disadvantage that each color combination is unique for each animal. All expression techniques are reserved for genetically well-characterized organisms with appropriate driver lines, and are thus not available for many classical model systems with already defined anatomical and functional connectivity. In addition, due to its specificity, genetic expression only ever targets subsets of neural circuits, and visualizing other circuit neurons depends on the availability of appropriate driver lines.

As an alternative, bath- and focal-applications, which are routinely used for drug and modulator administration, can be employed to deliver fluorescent dyes for bulk staining of cells [13], [26], [33], [70]. Various dyes are available that stain different cellular structures. While DAPI, for example, stains nuclear material in living tissue [71], exogenous applications of VSDs and calcium-sensitive dyes stain cell membranes and cytoplasm, respectively. While fluorometric calcium imaging requires membrane-permeant acetoxymethyl (AM) ester forms of the dye, many VSD associate with the neuronal membrane, revealing the outline of the stained neurons. Only cells within the application site will be stained, but within this area, the staining will lack specificity. One of the drawbacks of these dyes is that, for unknown reasons, not all dyes work in all systems [2], [72]. Potentially, chemical or mechanical differences between systems prevent the dyes from reaching the neural tissue. In particular in small invertebrate preparations, dyes have to either penetrate a nerve sheath in the periphery, or a ganglion sheath in more central regions of the nervous system. Often sheaths, glia cells and connective tissue impede dye penetration and must be removed surgically or enzymatically. During this potentially dangerous process, one risks mechanically damaging the nervous system while performing a microsurgery, along with more subtle effects related to changes in neural activity. We show that oscillations in the firing frequency of the proprioceptive neuron AGR in the STG disappear once the ganglion is desheathed, although all other features of neural activity remained intact. Thus, removing the sheath had subtle effects on the behavior of the system, potentially making the information acquired from such experiments less reliable. While the contribution of the ganglion sheath to neural activity is unclear in most systems, there are indications in several systems that removing the sheath might interfere with ionic balance [12] and mechanical properties [8]. We show that bath-application of the fluorescent VSD RH795 enhanced visibility of neural structures from the pre-stained condition in ganglia of three different species across two phyla without the need for removing the sheath. RH795 reliably stained somata, neurites and axons. In all ganglia cell bodies in several different layers of the ganglion could be located, despite the three dimensional structure of most ganglia. In the STG, which comprises only a single to a few layers of neurons, RH795 revealed many individually identifiable axons and neurites in a clarity never reported before in living tissue. In particular at the anterior end of the STG where axons were spatially spread out, individual axons of modulatory projection neurons that enter the STG [39] could be separated, in addition to identified motor neurons such as CD1 [41]. Intracellular recordings from these axons are traditionally performed in dark-field illumination [73], but our comparison of dark-field and fluorescence staining clearly reveals the superiority of the RH795 staining. It is thus conceivable that visually locating distinct axons before impalement will significantly increase recording success. Furthermore, our staining revealed axons that lay on top of cell bodies of other neurons, potentially impeding access to these neurons.

More importantly, RH795 stained axons and cell bodies in peripheral nerves through the sheath. In recent years, the modulation of axonal action potential propagation has attracted a lot of attention, demonstrating that axons are not mere cables that faithfully conduct information [74]. Rather, the intrinsic properties of peripheral axons are modified by neuromodulators via metabotropic receptors in the axon membrane. Axonal recordings are experimentally difficult and locating axons in the nerve is a major concern when multiple locations along the axon need to be recorded. Even in small invertebrates, motor and sensory axons are several centimeters long, enwrapped in a nerve sheath and packed into axon bundles [39]. Traditional methods for axon stainings, such as intracellular injection of dyes, only work at short distances, since diffusion of the dye inside of the neuron reduces dye concentration with distance. RH795 reliably stained axons through the nerve sheath without noteworthy residual staining of the sheath and revealed the fine details of individual axons and varicosities even in nerves with many (>60) axons. In nerves with fewer axons, almost all axons were immediately visible. For example, the axons of the two PD neurons, which are modulated by dopamine [75], were obvious in stainings of the *pdn*. In the small sensorimotor nerve *gpn*, we were able to locate the cell body of the gastro-pyloric receptor neuron, which has so far only been possible with retrograde labeling [42]. Furthermore, we were able to successfully localize AGR with RH795 staining through the sheath. We have shown that AGR activity changes when desheathing, so RH795 stainings will be particularly helpful in future experiments for locating the AGR soma for intracellular or optical recordings through the sheath.

In summary, RH795 staining allowed the comprehensive mapping of structural details in the STG never achieved in intact nerve tissue. Hence, RH795 is a good anatomical marker for determining cell body location as well as neuritic organization in living tissue.

### RH795 can be used to monitor neuronal activity through nerve sheaths

To understand neural circuit function, the detailed characterization of the activity of many, if not all, circuit neurons is needed [6], [9], [60], and ideally the location of the recorded neurons should be determined before or during the recording attempt. RH795 not only revealed anatomical features not observable in a non-stained ganglion, but also allowed us to record membrane potential changes through the sheath. While removing the sheath also enhances visibility in non-stained ganglia and facilitates access to neurons with microelectrodes, it comes with the caveats of possible damage to cells and activity. Most VSDs and calcium AM dyes are applied to desheathed ganglia [7], [9], [11], [13], [76] and, in the STG, do not appear to permeate the sheath (data not shown). We demonstrate that staining neurons through the sheath with RH795 not only retained the fine details of the neuronal activity, but also allowed us to record multiple neurons simultaneously and identify them using single-sweep (non-averaged) recordings. After spike-triggered averaging, the signal-to-noise ratio was high enough to detect the temporal synaptic dynamics of IPSPs between the LP and PD neurons. In general, the signal-to-noise ratio of optical imaging is lower than for direct measurements of the membrane potential with glass microelectrodes, although there have been significant improvements in the last decade [33], [35], [77]–[80]. There are more sources of optical noise than electrical (e.g. shot noise, dark noise and extraneous noise, [81], [82]). However, by averaging over multiple cycles, most of the noise can be removed.

Toxicity of the dyes is also an issue that needs to be taken into account. Most VSDs cause toxic effects over time, or affect neuronal properties that alter activity [83]. We have previously shown that RH795 has no discernable effect on the STG motor patterns and no apparent toxic influence when applied [26]. Prolonged illumination, however, can have deleterious effects on the motor pattern, limiting the duration of the imaging session [26], [38]. We also recognized a slow diminution of the staining over the course of several hours and along with it a diminishing signal-to-noise ratio. This effect was reversible by additional RH795 applications. Most importantly, RH795 allowed us to accurately monitor both the activity of fast pyloric and slow gastric mill neurons over an extended period of time. For the latter, we demonstrate the simultaneous recording of 4 GM neurons and Int1. We chose these particular neurons since their recording demonstrates the advantages of optical imaging over other recording techniques when it comes to separating the activities of neurons of the same type (such as the GMs) or recoding from small interneurons. The GMs are particularly interesting: they innervate the gm1 muscle [84], and intracellular recordings from individual GMs revealed similar synaptic input in all GMs [52]. Yet, GM neurons appear to vary in the projection pattern of their axons: The average number of GM axons on the *mvn* is 2.95 [53], indicating that subpopulations of GM neurons with different axonal projection patterns and possibly distinct activity patterns may exist. Nevertheless, our results indicate that, within the limitations of the optical imaging, GM membrane potentials were highly correlated during a VCN-elicited gastric mill rhythm [24], [47].

### Functional implications

Int1 and GM neurons are part of the gastric mill CPG [85] whose activity pattern is influenced by indirect feedback from the aforementioned proprioceptive neuron AGR. Our findings support the conclusion of Daur et al. [23] in that axonal modulation not only occurs spontaneously, but is reliably present when gastric mill rhythms are elicited. While changes in AGR firing frequency had been observed, they had never been correlated to the motor pattern before. We have previously shown that such small changes in AGR's spontaneous activity have significant effects on the motor pattern produced, as they determine the state of the neuromodulatory system that drives the gastric mill rhythm [23]. Thus, as AGR loses its oscillatory activity after desheathing, it is particularly important to keep the sheath intact while recording from Int1 and the GM neurons, which for all intents and purposes prevents multiple intracellular recordings. Extracellular recordings from motor nerves, on the other hand, cannot reveal Int1 activity, and do not allow separation of the 4 GMs from one another, a task easily solved by optical imaging with RH795.

With respect to AGR, our findings explain a previously published, conspicuous finding, namely that AGR activity appeared to be unaffected by other STG neurons despite the fact that it possesses putative postsynaptic structures on neurites in the STG [49]. In these experiments, desheathing the ganglion was necessary to locate AGR and its axons, but apparently reduced or abolished the postsynaptic response of AGR. We show that such a response is present in the non-desheathed ganglion and that applying RH795 does not influence the AGR response. Moreover, RH795 allows the localization of the AGR cell body without desheathing.

Because of the dual character of RH795 to function as an anatomical marker and as voltage indicator of membrane potential changes, there are multiple applications of this technique in addition to the examples demonstrated in this paper. In general, studies using semi-intact preparations may benefit from this approach as it can be difficult to maintain intracellular somatic recordings from neurons while the musculature is present and allowed to move. Depending on the species used, it may also be difficult to desheath ganglia in semi-intact preparations, given that nerves and connectives must remain intact.

This technique can also be applied to other ganglia in the STNS, like the commissural ganglia, which contains neurons that modulate the activity of STG neurons [21], [27] and are much less characterized. In particular for preliminary studies, using RH795 may be an expeditious technique to acquire information about populations of modulatory cells, and to characterize the response and location of these neurons.

In summary, RH795 provides a relatively unique tool for recording many neurons simultaneously in living tissues without the need for removing neural sheaths, and has a plethora of other potential applications not yet investigated.

## Supporting Information

Movie S1 **Simultaneous single-sweep optical recordings through the ganglion sheath of 3 pyloric neurons (IC and two PDs, compare to** **Figure 5A** **).** Left: Fluorescence change over time of three regions of interest, as marked in the photo on the right. Right: Changes in fluorescence are represented as changes in the color intensity. Intense colors represent a depolarization of the membrane potential and correspond to the point in time indicated by the vertical line in the left diagram. For better visualization of the pyloric details, the video speed is decreased by a factor of two.
(AVI)

Movie S2 **Simultaneous single-sweep optical recordings through the ganglion sheath of 5 gastric mill neurons (Int1 and 4 GMs, compare to** **Figure 7** **).** Left: Fluorescence change over time of 5 regions of interest, as marked in the photo on the right. Intense colors represent a depolarization of the membrane potential and correspond to the point in time indicated by the vertical line in the left diagram. For better visualization of alternating gastric mill neuron activities, the video speed is increased by a factor of two.
(AVI)

Raw Data S1 **Raw data for** **figures 4** **,** **5** **,** **6** **, and** **7** **.**
(XLSX)
